# To establish a talent pool for global health in China: from political will to action

**DOI:** 10.1080/16549716.2018.1489603

**Published:** 2018-08-14

**Authors:** Jiye Fu, Chu Jiang, Juguang Wang, Yan Xing

**Affiliations:** a Department of Immunization Program, Beijing Haidian District Center for Disease Control and Prevention, Beijing, People’s Republic of China; b Beijing Haidian District Center for Disease Control and Prevention, Beijing, People’s Republic of China

**Keywords:** Global health, human resource for health, China

## Abstract

Recently, China has played a more and more important role in global health, mainly by improving health outcomes of its own population nationwide and participating international health activities. In addition, China has participated in a dozen of international organizations, which all contained health domains, particularly the Belt and Road Initiative with an ambitious goal to improve health of the people in the countries alongside closely partnered with the World Health Organization. All these highlight the need of human resource for global health at the national level. The National Health and Family Planning Commission translated this political will into action – that a talent pool candidate for global health will be established in China. The establishment of the talent pool would be of great significance for China’s engagement in global health activities. However, much work, such as training, collaboration, and innovation, etc., remains to be done in the future. Based on the successful practice, China can share lessons learned to other countries.

Recently, China has played a more and more important role in global health. By exerting deepening health transform nationwide since 2009, improving health insurance coverage, and increasing investment in health, China has improved health outcomes of its population (about 18% of the world’s population) [1,2]. On the other hand, since 1960s, China has actively engaged in international health activities, including construction of health facilities, health-related donations, and dispatches of health workers in support of local medical care and communicable diseases control, mainly in resource-limited countries and regions [3,4]. In addition, China has participated in a dozen of international organizations, such as Asia-Pacific Economic Cooperation (APEC), Shanghai Cooperation Organization, and South-South cooperation, etc., which all contained cooperation in health dimensions [5]. Furthermore, the Chinese President Xi Jinping’s proposal of the Belt and Road Initiative in 2013 even took health as the core of development, with an ambitious goal to build a healthy Silk Road closely partnered with the World Health Organization (WHO) to improve the health of people in countries alongside [6,7]. All these highlight the importance and urgent need of human resource, the critical component of health system, for global health at the national level in China.

However, China lacks a formal pool of on-call, well-trained responders that could be rapidly mobilized an external period and in large numbers. Since 1963, China has built more than 100 health facilities with an estimated 23,000 health workers dispatched overseas with health aid [2]. Medical teams deployed to countries in Africa or in other regions were selected from public hospitals at the subnational level in the absence of public health workers until the health teams dispatched to respond to Ebola outbreak in West Africa in 2013 [8]. For the short-term health affairs, health workers including clinicians, nurses, public health professionals, and health officials were selectively gathered from corresponding departments to compose an interim team, which then would be deployed to commit health activities under the leadership of National Health and Family Planning Commission (NHFPC), the highest leader in health system in China. For example, two teams, involving nine staff members from Chinese Center for Disease Control and Prevention (CDC), Jilin and Gansu Provincial CDC, and Beijing Ditan Hospital, etc., were sent to Madagascar to assist local authorities in halting plague outbreak in October 2017 [9]. For the long-term international health activities, such as delivery of health aid activities in African countries, nearly each Chinese province was twinned with one country in Africa [2]. However, short mobilizations and frequent staff rotation in the field also disrupted development of long-standing relationships and continuity of response. On the other hand, longer mobilizations of a large workforce could hamper staff members’ regular duties [10]. Additional challenges included identifying staff with the appropriate technical skills and foreign language abilities, which were well prepared for the austere conditions. Thereby it is critical to establish a talent pool for health at the national level in China.

In October 2016, China released the Healthy China 2030 Strategies [11], and unveiled its National 13th Five-Year Development Plan on Human Resource for Health in January 2017 [12], planning to build a health team at the national level. In accordance with these plans, the NHFPC translated this political will into action. On 8 August 2017, the NHFPC issued a notification [13] that a talent pool candidate for global health would be established in China, encouraging nationwide clinicians, public health workers, health managers, and experts from medical universities to participate. Consequently, in addition to a foreign language test implemented in November 2017, the applicants will pass through expertise interview combined knowledge, skills, and abilities required to performing a task effectively, which will be launched in 2018. Moreover, those with qualification by both foreign language test and interview will be eventually recruited in the talent pool.

Several factors, including substantially increased investment [1], strengthening collaboration across health and non-health agencies [2,5], and a growing number of researchers in global health [14,15], particularly in the past decade, make such a talent pool viable in China. The total health expenditure in China has increased more than 20-fold during the past two decades [16], accounting for 6.2% of its gross domestic product in 2016 [17], and the investment in global health research has risen accordingly. On the other hand, collaboration mechanism across health and non-health agencies have been established since outbreak of SARS in China in 2003 [18], which operated effectively in response to the H1N1 influenza pandemic in 2009 and was strengthened in combating emergencies of human infection with H7N9 influenza virus in 2013 and in prevention and control of other major communicable diseases [19]. Additionally, China has made huge achievements in human resource for health since 1978, with the number of health professionals growing from 5.53 million in 2009 to 8.45 million in 2016 [17]. Furthermore, the Consortium of Chinese Universities on global health has been established since 2013 [14,15], aiming to promote global health development in China by sharing knowledge and resource, focusing on research and education in collaboration with researchers from international universities, and facilitating Chinese participation in global health activities abroad [2,15].

The establishment of the talent pool would be of great significance for China’s engagement in global health. When major health threats, such as emerging infectious diseases cross-border and recovery of natural disasters, occur in resource-limited countries, wide range of technical expertise would be required to address these threats. Requested assistance from within NHFPC and from external partners, the members of the pool will be gathered and commit the health assistance under the leadership of the NHFPC. However, much work remains to be done to make sure the members implement a task effectively.

First, training. The personnel in the absence of an oversea health career track may lack appropriate health or diplomacy skills. Therefore, systematic trainings, including not only knowledge, skills, and capabilities in expertise domains, language and communications, relevant laws or regulations, such as International Health Regulations (2005) (IHR), but also interprofessional education, epidemic preparedness, and emergency response, are warranted. The NHFPC is responsible for the training in coordination with other Chinese government agencies, such as ministries of education, commerce, foreign affairs, science and technology, and will provide opportunities for the members of the talent pool to participate in international health activities, such as international conferences, and conduct research with international partners.

Second, collaboration. The collaboration would not be only limited to members within the network, but be also with local health departments, healthcare provider systems, and international organizations. Only by seamless collaboration, the staff from different agencies with different background can they respond successfully and effectively, when facing emergency situations, for instance, natural disasters recovery and communicable diseases outbreak control. In major health activities overseas, especially in response to Ebola in West Africa during 2013–2015, China has realized the importance of cooperation and collaborations with international organizations. In reality, China has launched the new Country Cooperation Strategies 2016–2020 with WHO, aiming to strengthening the cooperation between China and WHO, with six health priorities including enhancing China’s contribution in global health [8]. On the other hand, the cooperation between China and other international partners was also launched, including EU CDC [20], US CDC [21,22], UNICEF, and health ministries of four BRICS countries [23], sharing concerns about cross-border infectious disease threats, and combating other major health threats, such as ageing population, and conducting medical research.

Third, innovation. Technological innovation (e.g. social media in dissemination of health knowledge and in data collection, digital mapping in visualization of geographic distribution of communicable and non-communicable diseases cases, and gene technology in diagnosis of disease, etc.) has played a key role in today’s health activities. And unprecedented growth in mobile telecommunications, internet, and gene techniques not only made these innovations viable in low- and middle-income countries but also can make health care and public health interventions more affordable, more accessible, and more effective. The staff members from the pool should fully utilize these innovations in cooperation with the local authorities.

Finally, sustainability. The talent pool should be renewed every three or four years. Certification should be implemented to assess whether the members qualified or not. Members should be terminated of eligibility for any of, including but not limited to, followings: unfit due to health condition, unable to involve in committed activities, or failure to comply with laws or regulations, etc. In addition, newly qualified health personnel are encouraged to participate. On the other hand, it is critical to explore new investment mechanisms to make the government’s efforts in global health more effective and efficient in addition to be financed by the health aid budget in NHFPC, which is responsible for dispatching medical teams.

The establishment of the talent pool in China will pave the way for international cooperation to protect people from health threats and to ensure health security. Although the experiences may not be replicated, China can share its lessons learned to other countries based on the successful practices [24]. Despite of challenges ahead, China will continue its active engagement in global health to improve health of both its and worldwide people in collaboration with WHO and other international partners, and to make progress towards goal of the universal health coverage.

## Paper context

Recently, China has played a more and more important role in global health, mainly by participating international health activities and implementing health cooperation. These highlight the need of human resource for global health at the national level. A talent pool for global health will be established in China in 2018. Despite of great significance for China’s engagement in global health activities, much work, such as training, collaboration, and innovation, etc., remains to be done.
